# Non-invasive ventilation indication for critically ill cancer patients admitted to the intensive care unit for acute respiratory failure (ARF) with associated cardiac dysfunction: Results from an observational study

**DOI:** 10.1371/journal.pone.0234495

**Published:** 2020-06-10

**Authors:** Colombe Saillard, Damien Mallet, Laurent Chow-Chine, Magali Bisbal, Marion Faucher, Antoine Sannini, Djamel Mokart

**Affiliations:** 1 Hematology Department, Institut Paoli Calmettes, Marseille, France; 2 Polyvalent Intensive Care Unit, Department of Anesthesiology and Critical Care, Institut Paoli Calmettes, Marseille, France; Nazareth Hospital, UNITED STATES

## Abstract

**Background:**

Acute respiratory failure (ARF) is a life-threatening complication in onco-hematology patients. Optimal ventilation strategy in immunocompromised patients has been highly controversial over the last decade. Data are lacking on patients presenting with ARF associating isolated cardiac dysfunction or in combination with another etiology. The aim of this study was to assess prognostic impact of initial ventilation strategy in onco-hematology patients presenting ARF with associated cardiac dysfunction.

**Methods:**

We conducted an observational retrospective study in Institut Paoli-Calmettes, a cancer-referral center, assessing all critically ill cancer patients admitted to the ICU for a ARF with cardiac dysfunction.

**Results:**

Between 2010–2017, 127 patients were admitted. ICU and hospital mortality were 29% and 57%. Initial ventilation strategy was invasive mechanical ventilation (MV) in 21%. Others ventilation strategies were noninvasive ventilation (NIV) in 50%, associated with oxygen in 21% and high flow nasal oxygen (HFNO) in 29%, HFNO alone in 6% and standard oxygen in 23%. During ICU stay, 48% of patients required intubation. Multivariate analysis identified 3 independent factors associated with ICU mortality: SAPSII at admission (OR = 1.07/point, 95%CI = 1.03–1.11, p<0.001), invasive fungal infection (OR = 7.65, 95%CI = 1.7–34.6, p = 0.008) and initial ventilation strategy (p = 0.015). Compared to NIV, HFNO alone and standard oxygen alone were associated with an increased ICU mortality, with respective OR of 19.56 (p = 0.01) and 10.72 (p = 0.01). We realized a propensity score analysis including 40 matched patients, 20 in the NIV arm and 20 receiving others ventilation strategies, excluding initial MV patients. ICU mortality was lower in patients treated with NIV (10%), versus 50% in the other arm (p = 0.037).

**Conclusion:**

In onco-hematology patients admitted for ARF with associated cardiac dysfunction, severity at ICU admission, invasive fungal infections and initial ventilation strategy were independently associated with ICU mortality. NIV was a protective factor on ICU mortality.

## Introduction

Acute respiratory failure (ARF) is the most frequent and severe life-threatening complication in onco-hematology patients, and remains the first reason for intensive care unit (ICU) admission in cancer patients [1]. ARF occurs in 5% of patients with solid tumors, 15% of patients with hematological malignancies and up to 50% in cancer neutropenic patients [2]. Nearly 15% of cancer patients developing ARF require admission to the ICU [3] and intensivists will be increasingly asked to manage these patients given the growing incidence of cancer and population aging. ARF requires a careful and systematic diagnostic strategy, which is now well established [2].

In cancer patients admitted to the ICU for ARF, the need for intubation and invasive mechanical ventilation (MV) is associated with particularly high mortality rates, reaching 70% [4, 5]. Actual mortality rate in hematology patients admitted to the ICU for ARF is 43%(1). The main prognostic factors are MV requirement [1, 6, 7], ARF etiology [1,8], poor performance status, delayed ICU admission [9], associated organ dysfunctions [1] and allogeneic hematopoietic stem cell transplantation patients [10].

Optimal ventilation strategy in immunocompromised patients has been highly controversial over the last decade. The choice between standard oxygen, non-invasive ventilation (NIV), mechanical ventilation and high-flow nasal oxygen (HFNO) remains a matter of debate [11–15]. In the early 2000s, mortality of patients needing MV reached 90% [3, 16]. Survival benefits were reported with NIV [3] and appeared as an efficient alternative to invasive MV, even though delayed intubation after NIV failure was associated with higher mortality[4]. Consequently, NIV was largely used as the first-line ventilation strategy [6, 17, 18]. However, most recent studies did not confirm these results [19]. Survival of cancer patients admitted to ICU improved, even for patients receiving MV [6, 20, 21]. Therefore, survival benefits from NIV could either be harder to demonstrate or may have been balanced by changes in ventilation strategy selection [18, 22]. Strategies can differ according to ARF etiology and severity. NIV has been validated in acute cardiac pulmonary edema (ACPE) and acute exacerbations of chronic obstructive pulmonary disease (COPD) with decreased intubation and mortality rates [23]. Data are lacking on patients presenting with ARF associating isolated cardiac dysfunction or in combination with another etiology. The aim of this study was to assess prognostic impact of initial ventilation strategy in onco-hematology patients presenting ARF with associated cardiac dysfunction.

## Material and methods

### Patients’ selection

We conducted an observational study from 2010 to 2017 in our institution (Paoli-Calmettes Institute, Marseille, France), a 211-bed cancer referral center. We retrospectively analyzed all cancer patients admitted to the ICU for ARF with cardiac dysfunction using our ICU information system (Metavision; iMDsoft, Tel Aviv, Israel). Briefly, in our institution ICD-10-CM diagnosis coding is not yet sufficiently exhaustive for cardiac dysfunctions such as left ventricular failure (ICD-10-CM code: I 50.1), heart failure (I.50), acute diastolic heart failure (I.50.31) while the diagnosis of acute respiratory failure (J96.0) or acute pulmonary edema (J81.0) were more frequently notified. Consequently, the search for patients likely to be included was first carried out using our ICU information system in which we extracted all the patients treated with respiratory support (standard O2, HFNO, NIV and MV) during the chosen period. Secondly, we analyzed all the hospitalization reports of these patients and selected the patients with confirmed ARF. Thirdly, we confirmed and completed this first selection of patients by analyzing all the echocardiographic data to check up cardiac involvement in ARF. Finally, the last step consisted of completing this selection of patients by using the coding information of hospitalization reports using the ICD-10-CM diagnosis codes for the following pathologies: acute respiratory failure (J96.0), acute pulmonary edema (J81 .0), left ventricular failure (I 50.1), heart failure (I.50), acute diastolic heart failure (I.50.31).

The inclusion criteria were as follows: age ≥ 18 years, patient with solid cancer or hematological malignancy, patient presenting with ARF associated with cardiac dysfunction at ICU admission. The exclusion criteria were as follows: absence of cancer, ARF without cardiac dysfunction at ICU admission, age <18 years, pregnant or lactating woman. All data were fully anonymized before we accessed them. This study was approved by our “Institutional Review Board” (Institut Paoli Calmettes, IPC2019-003), which waived the requirement for informed consent. The methodology adheres to the STROBE statement [24].

### Definitions and data collection

Clinical and biological data were retrospectively collected. ARF was defined as a need for oxygen greater than 6 L/min to maintain peripheral capillary oxygen saturation (SpO2) above 95% or symptoms of respiratory distress (tachypnea >30/min, intercostal recession, labored breathing, and/or dyspnea at rest). The following data were collected during the ICU stay: age, gender, underlying malignancy, disease status, neutropenia (absolute neutrophil count < 0.5 G/L), chronic health status as evaluated by the Knaus scale, comorbidity according to Charlson comorbidity index, severity of illness scores using Simplified Acute Physiology Score II (SAPS II) and sepsis-related organ failure assessment score (SOFA) at admission, biological data including microbial documentation, computed tomography (CT) scan patterns, ARF etiology, therapeutic interventions, length of ICU stay, ICU mortality and hospital mortality. Sepsis and septic shock were defined as previously described [25, 26]. Succinctly, sepsis was defined by a suspected infection associated with an acute increase of ≥ 2 SOFA points. Septic shock was defined by the association of sepsis with vasopressor therapy needed to elevate MAP ≥65 mm Hg and lactate level >2mmol/L despite adequate fluid resuscitation. We also collected the history of exposure to cardiotoxic drugs and the number of red blood cells and platelet transfusions 72 hours before ICU admission.

Hemodynamic evaluation using echocardiography was routinely performed in all patients by experienced physicians using a General Electric Vivid i machine (General Electric Comp., Boston, USA) using the standard parasternal and apical views. The following measurements were collected: left ventricle (LV) end-diastolic volume and LV end systolic volume using the biplane modified Simpson’s rule from which LV ejection fraction (LVEF) was calculated; the peak mitral inflow E and A velocity waves on pulsed-Doppler (E/A ratio), the diastolic e’ peak velocity and the LV filling index E/e’ ratio and tricuspid annular plane systolic excursion (TAPSE). As previously described [25, 26], systolic dysfunction was defined by LVEF ≤50%, diastolic dysfunction by e′ ≤8 cm s^-1^, left ventricular dysfunction (LVD) by the presence of impaired LVEF and/or impaired diastolic dysfunction, right ventricular dysfunction (RVD) by a TAPSE < 16 mm or a RV dilatation (RV/LV ratio > 0.6) or a systolic pulmonary arterial pressure >45 mmHg. ARF with cardiac dysfunction was defined as an ARF associated with LVD and/or RVD. A mixed ARF was defined as an ARF with cardiac dysfunction in combination with another etiology.

Sepsis was classified as microbiologically documented in case of fever with pathogens identified. Invasive pulmonary aspergillosis was defined according to the 2008 EORTC criteria. Only proven and probable aspergillosis diagnoses were included. Viral pneumonia was diagnosed if a virus was detected by viral culture or PCR in BAL fluid, blood or nasopharyngeal specimens with clinical features consistent with viral pneumonia. *Candida* spp. in BAL fluid or sputum was interpreted as a colonization. Bacteremia was diagnosed if a bacterial pathogen was isolated in a least one blood culture.

According to clinical presentation, severity and ARF etiology, the initial ventilation strategy consisted of MV, NIV (associated with standard oxygen or HFNO), HFNO (alone or in combination with NIV) or standard oxygen alone. Other medical treatments consisted of diuretics, vasodilators, antimicrobial treatments according to standard recommendations and organ support such as vasopressors and renal replacement therapy (RRT).

### Statistical analysis

Sample size calculation: There is currently no data in the literature regarding mortality of cancer patients with ARF with associated cardiac dysfunction. In the Masip meta-analysis (23), the mortality of patients (general population) with cardiogenic edema treated with NIV was 11%, while the ICU mortality of cancer patients with ARF was around 40% [15]. Our hypothesis was that the mortality of cancer patients with cardiogenic edema treated with NIV is greater than 10%, around 15% (personal data), due to the frequently mixed nature of this clinical picture and comorbidities in cancer patients. Thus, based on a 15% ICU mortality rate in the NIV group and a 40% ICU mortality rate in the non NIV group, with an α risk set at 5% and a 90% power for demonstrating the superiority for the NIV use, we needed to include at least 106 patients.

All data are presented as rates (percentage) for qualitative variables and medians (25th-75th percentiles) for quantitative variables. Characteristics of patients were compared across the groups of ICU survivors and decedents by using Fisher’s exact test and Wilcoxon rank-sum test. We performed logistic regression analyses to identify variables independently associated with ICU mortality, as measured by the estimated odds ratio (OR) with 95% confidence interval (95% CI). Variables yielding *p* lower than 0.05 in the bivariate analyses were entered into a backward stepwise logistic regression model where ICU mortality was the outcome variable of interest. Last, we forced variables of clinical interest that may be suspected to be associated with outcome into the final model. The Hosmer-Lemeshow test was used to check goodness-of-fit of the logistic regression. All tests were two-sided, and *p* values lower than 0.05 were considered statistically significant. Analysis was performed using SPSS, IBM^Ⓡ^ SPSS version 16.0 (IBM Corp., Armonk, USA)

A propensity score-based approach was used to limit bias of between-group comparison to assess the impact of NIV compared to other ventilation strategies on ICU mortality. Patients receiving immediate MV were excluded of this analysis. Patients were matched according a 1:1 ratio, in the NIV arm and in the “other ventilation strategy” arm. The propensity score was defined as the probability that a patient with specific baseline characteristics received NIV. Then, two patients with identical propensity score value but in the two different treatment groups can be considered as comparable. Matching on the propensity score has been shown as one of the most efficient methods for treatment effect assessment. We computed the propensity score using logistic regression based on baseline characteristics known to be associated with mortality (underlying malignancy, age, gender, SAPSII at admission, SOFA score at admission, neutropenia, allogenic HSCT recipients, ARF etiology, vasodilatators, diuretics, vasopressors use). Standardized differences were used to compare balance in baseline covariates between the two groups. A 1:1 matching algorithm without replacement was used within a given range of 0.20 standard deviations of the logit of the estimated propensity score. Final analyses on the matched dataset were performed using a conditional logistic regression on the paired observations. All tests were two-sided, and p values lower than 0.05 were considered statistically significant.

## Results

### Patients’ characteristics at ICU admission and during ICU stay

**Fig 1:**
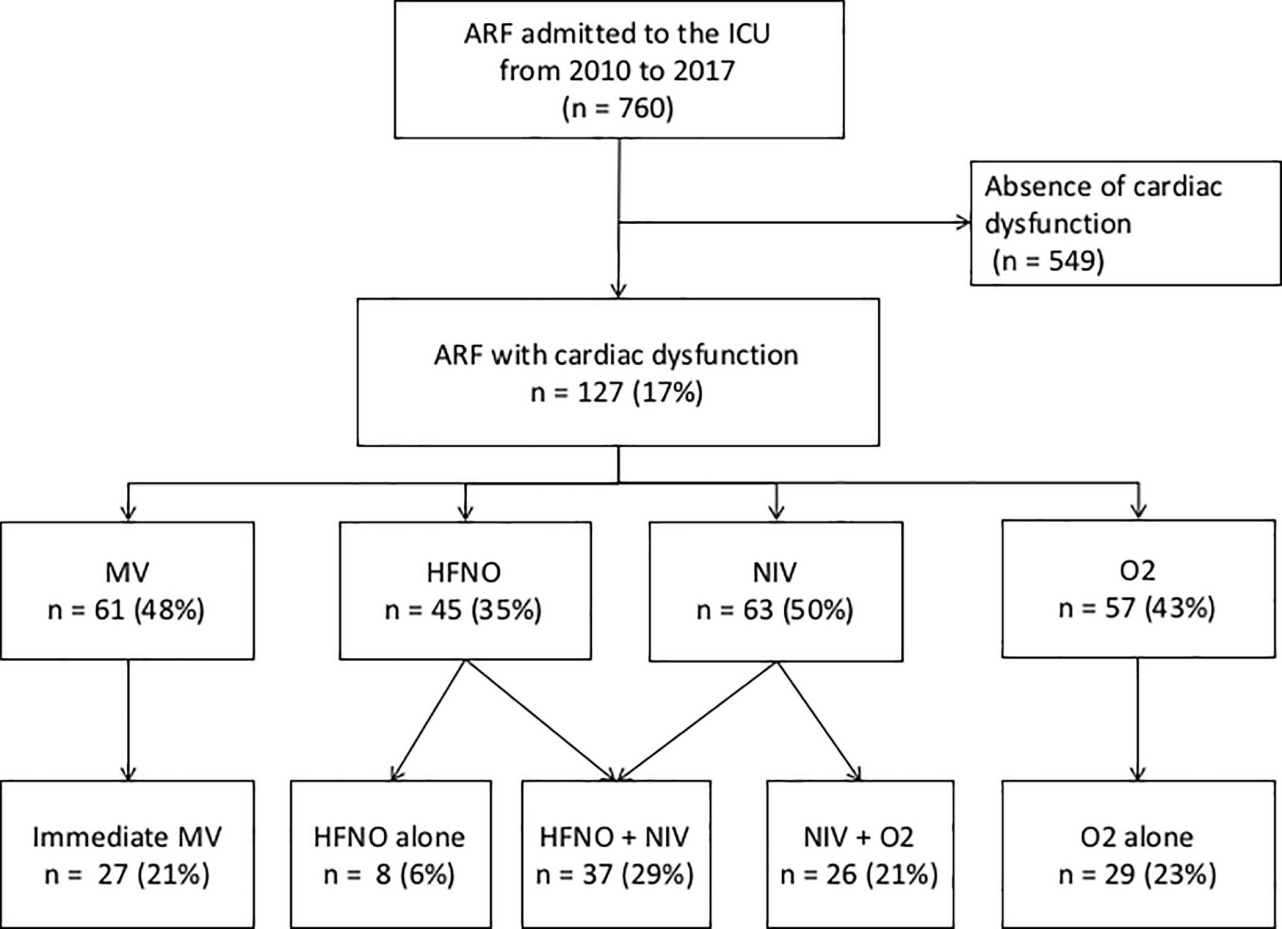
Flow chart of patients’ selection and ventilation strategy. ARF: acute respiratory failure, HFNO: high flow nasal oxygen, ICU: intensive care unit, MV: mechanical ventilation, NIV: non-invasive ventilation, O2: standard oxygen.

Between 2010 and 2017, 760 patients were admitted to the ICU for ARF. One-hundred twenty-seven patients (17%) had a diagnosis of ARF with cardiac dysfunction (Fig 1). Main characteristics of patients at ICU admission are reported in Table 1. Median age was 66 (59–74). Median Charlson comorbidity index was 4 (2–8), the main comorbidities being COPD (11%), diabetes mellitus (18%), chronic cardiac failure (17%), coronary heart disease (12%) and arterial hypertension (46%). Hematological malignancies and solid tumors represented 76 and 48 patients respectively. Fourteen-percent of patients were newly diagnosed, 23% were in complete or partial response and 63% had a progressive disease. Allogeneic and autologous hematopoietic stem cell transplantation recipients represented 27% and 7% of patients respectively. Median SAPSII and SOFA score at ICU admission were 50 (42–64) and 8 (5–11) respectively. Eighty-two percent of patients were septic at ICU admission. Sepsis was microbiologically documented in 32%. Antimicrobials were used in 81% of patients. Other organ failures were represented by hemodynamic and renal failure. Thirty four percent of patients required vasopressors at ICU admission and 48% during ICU stay. Forty three percent of patients were admitted to the ICU with acute kidney injury and 21% required renal replacement therapy. Median ICU length was 4 days (2–10). ICU length was 4 days (2–10). ICU mortality and hospital mortality rates were 29% and 57% respectively. Among all patients treated initially with NIV at ICU admission (n = 63) the ICU mortality rate was 16% (n = 10) compared to 42% (n = 27) in patients not initially treated with NIV, p = 0.001.

**Table 1:** Characteristics of patients at ICU admission and during ICU stay.

	All patients (n = 127)	ICU non survivors (n = 37)	ICU survivors (n = 90)	*p*
Age (years)	66 (59–74)	63 (58–71)	67 (59–74)	0.3
Gender (female/male) (%)	68/59	20/17	48/42	1
Charlson comorbidity index	4 (2–8)	4 (2–9)	4 (2–6)	0.3
Comorbidity				
COPD	14 (11)	6 (16)	8 (9)	0.2
Diabetes mellitus	23 (18)	5 (14)	18 (20)	0.5
Chronic cardiac failure	21 (17)	8 (22)	13 (14)	0.3
Coronary heart disease	15 (12)	1 (3)	14 (16)	0.06
Arterial hypertension	59 (46)	17 (46)	42 (47)	0.9
Underlying malignancy				
Hematological malignancies	76 (60)	23 (62)	53 (58)	0.9
AML/MDS	40 (31)	16 (42)	24 (26)	0.3
Lymphoma	16 (13)	4 (11)	12 (13)	0.8
Myeloma	10 (8)	1 (3)	9 (10)	0.3
CLL	4 (3)	1 (3)	3 (3)	0.7
Others (PMN, AA, ALL)	6 (5)	1 (3)	5 (6)	0.7
Solid tumors	48 (38)	14 (38)	34 (38)	0.8
Unknown	3 (2)	0 (0)	3 (2)	0.7
HSCT recipients				
Allogenic HSCT	34 (27)	11 (30)	23 (26)	0.7
Autologous HSCT	9 (7)	2 (5)	7 (8)	1
Disease status				
Complete or partial response	29 (23)	5 (14)	24 (27)	0.2
Progression	80 (63)	27 (73)	53 (59)	0.2
Not available (newly diagnosed)	18 (14)	5 (13)	13 (14)	0.1
Neutropenia	41 (32)	13 (35)	28 (31)	0.7
Severity scores at ICU admission				
SAPSII	50 (42–64)	64 (49–72)	46 (40–58)	**<0.001**
SOFA score	8 (5–11)	10 (8–11)	7 (5–10)	**0.001**
Biological parameters at ICU admission				
Lactates (mmol/L)	2.5 (1.5–4.8)	3.4 (1.8–4.9)	2.3 (1.4–3.8)	0.09
Troponine (ng/mL)	0.14 (0.05–0.9)	0.14 (0.06–0.98)	0.15 (0.05–0.78)	1
Procalcitonine (μg/L)	3.3 (0.5–15.8)	5.9 (1.5–23.8)	1.46 (0.41–9.61)	0.06
BNP (ng/mL)	1153 (555–2721)	1267 (508–3082)	1139 (606–2467)	0.4
Creatinine (μmol/L)	108 (72–157)	111 (78–170)	104 (68–153)	0.6
Total bilirubin (μmol/L)	21 (12–36)	22 (12–34)	21 (12–38)	0.8
Platelets (giga/L)	79 (32–217)	58 (21–109)	114 (35–245)	**0.03**
Hemodynamic failure				
Vasopressors				
At ICU admission	43 (34)	17 (46)	26 (29)	0.06
During ICU stay	61 (48)	29 (78)	32 (36)	**<0.001**
Noradrenaline	47 (37)	22 (59)	25 (28)	**0.001**
Dobutamine	20 (16)	8 (22)	12 (13)	0.24
Adrenaline	30 (24)	20 (54)	10 (11)	**<0.001**
Renal failure				
AKI at ICU admission	54 (43)	18 (49)	36 (40)	0.4
RRT	27 (21)	11 (31)	16 (18)	0.1
Infection				
Sepsis at ICU admission	104 (82)	33 (89)	71 (79)	0.3
Microbiologically documented	40 (32)	14 (38)	26 (29)	0.3
Bacteria	34 (27)	10 (27)	24 (27)	0.8
Gram-negative bacilli	35 (28)	9 (24)	26 (29)	0.6
Gram-positive cocci	15 (12)	6 (16)	9 (10)	0.3
Multi-resistant pathogens	11 (9)	3 (8)	8 (9)	0.9
Fungi	12 (9)	7 (19)	5 (6)	**0.04**
*Aspergillus*	7 (6)	5 (14)	2 (2)	
*Pneumocystis Jiroveci*	2 (2)	1 (3)	1 (1)	
*Candida*	2 (2)	1 (3)	1 (1)	
*Fusarium*	1 (1)	1 (3)	0 (0)	
Virus	6 (5)	1 (3)	5 (6)	0.8
Antibiotherapy				
Absence	24 (19)	5 (14)	19 (21)	0.3
Empirical	64 (50)	19 (51)	45 (50)	0.9
Appropriate	38 (30)	13 (35)	25 (28)	0.4
Inappropriate	1 (1)	0 (0)	1 (1)	1

AA: aplastic anemia, AKI: acute kidney injury, ALL: acute lymphoid leukemia, AML: acute myeloid leukemia, ARF: acute respiratory failure, BNP: brain natriuretic peptide, CLL: chronic lymphoid leukemia, COPD: chronic obstructive pulmonary disease, HSCT: hematopoietic stem cell transplantation, ICU: intensive care unit, MDS: myelodysplastic syndromes, MPN: myeloproliferative neoplasms, RRT: renal replacement therapy, SAPSII: simplified acute physiology score, SOFA: sepsis-related Organ Failure Assessment.

### ARF characteristics (Table 2)

Median PaO2/FiO2 at ICU admission was 219 (133–327). Almost half patients (48%) received cardiotoxic chemotherapy in the last 3 months. Respectively 40% and 32% of patients required red blood cells and platelets transfusion in the 72 hours preceding ARF onset. All patients presented ARF with cardiac dysfunction. The main ARF etiologies were cardiogenic edema with LVD in 116 patients (91%), RVD in 31 (24%), sepsis in 104 (82%) including 58 (46%) infectious pneumonia, pulmonary embolism in 6 (5%), and pleural effusion in 12 (9%). In 23 (18%) patients, cardiac dysfunction was the only cause of ARF, in 104 (82%) patients ARF etiologies were mixed. Median lactates, troponin and brain natriuretic peptide levels were 2.5 mmol/L (1.5–4.8), 0.14 ng/mL (0.005–0.9) and 1153 ng/mL (555–2721) respectively. Chest CT-scan revealed interstitial pattern in 25% and ground glass opacities in 17%. The four quadrants were involved in 55% of patients. Initial ventilation strategy at ICU admission consisted of immediate MV for 21% of patients. Others patients received NIV in 50%, which was associated with oxygen in 21% and HFNO in 29%, HFNO alone in 6% and standard oxygen alone in 23%. During ICU stay, 48% of patients required intubation and MV (Fig 1). Among the 100 patients who were not treated initially with MV, subsequent MV treatment was used in 1 (12.5%) patient out of the 8 patients initially treated with O2 alone was, in 18 (62.1%) patients out of the 29 initially treated with HFNO alone, in 5 (19.2%) patients out of the 26 initially treated with NIV + O2 and in 12 (32.4%) patients out of the 37 patients initially treated with NIV + HFNO. For those patients, the delay between initial strategy and the use of MV was 1 (0–3) day, 3 (3–3) days, 0 (0–13.5) days and 1.5 (0–6.75) days, respectively.

**Table 2:** Characteristics of acute respiratory failure.

	All patients (n = 127)	ICU non survivors (n = 37)	ICU survivors (n = 90)	*p*
Cardiotoxic chemotherapy in the 3 months	61 (48)	17 (46)	44 (49)	0.8
Transfusion in the last 72 hours				
RBC	51 (40)	12 (32)	39 (43)	0.3
Platelets	41 (32)	13 (35)	28 (31)	0.6
ARF diagnosis				
ARF with LVD	116 (91)	30 (80)	86 (97)	0.01
ARF with RVD	31 (24)	11 (31)	20 (22)	0.4
Sepsis	104 (82)	33 (89)	71 (79)	**0.3**
Infectious pneumonia	58 (46)	19 (51)	39 (44)	0.3
Pulmonary embolism	6 (5)	3 (8)	3 (3)	0.6
Pleural effusion	12 (9)	5 (14)	7 (9)	0.4
Atelectasia	1 (1)	0 (0)	1 (1)	0.3
Others	22 (17)	6 (16)	16 (18)	0.8
Pa02/Fi02	219 (133–327)	187 (128–276)	239 (121–329)	0.4
CT scan parameters (/52 patients)				
Interstitial pattern	32 (25)	7 (19)	25 (28)	**0.02**
Ground glass opacities	22 (17)	10 (27)	12 (13)	0.2
Number of involved quadrants (/115 patients)				0.07
0	7 (6)	2 (6)	5 (6)	
1	1 (1)	0 (0)	1 (1)	
2	37 (30)	15 (44)	22 (24)	
3	10 (8)	3 (9)	7 (8)	
4	67 (55)	14 (41)	53 (59)	
Echocardiography parameters at ICU admission				
Systolic dysfunction				
No (LVEF >50%)	32 (25)	9 (24)	23 (26)	1
Impaired (40% ≤ LVEF≤ 50%)	44 (35)	14 (38)	30 (33)	0.7
Highly impaired (LVEF <40%)	39 (31)	11 (38)	21 (33)	0.6
Diastolic dysfunction (e’ ≤ 8cm/sec)	44 (35)	13 (35)	31 (34)	1
E/A	1.6 (1–2.3)	1.25 (0.9–1.7)	1.65 (1.1–2.5)	0.1
E/e’	11.4 (8.4–16)	11 (8.5–14.9)	12.4 (8.4–15.9)	0.9
Right ventricular dysfunction	31 (24)	11 (31)	20 (22)	0.4
Systolic pulmonary arterial pressure >45 mmHg	8 (6)	3 (8)	5 (6)	0.7
RV dilatation	4 (3)	1 (3)	3 (3)	0.8
TAPSE < 20 mm	19 (15)	6 (16)	13 (14)	0.8
Ventilation strategy at ICU admission				
MV				
Immediate MV	27 (21)	8 (22)	19 (21)	0.9
MV during ICU stay	61 (48)	31 (84)	30 (33)	**<0.001**
NIV	26 (21)	2 (5)	24 (27)	**0.007**
HFNO alone	8 (6)	4 (11)	4 (4)	0.3
NIV + HFNO	37 (29)	8 (22)	29 (32)	0.3
Standard oxygen alone	29 (23)	15 (41)	14 (16)	**0.004**
Other treatments				
Diuretics	101 (80)	22 (59)	79 (88)	**0.001**
Hydric balance at 48 hours (L)	-1.3 (-3.6;1.6)	1.3 (-0.9;3.1)	-1.9 (-4;0.6)	**<0.001**
Vasodilators	50 (39)	8 (22)	42 (47)	**0.01**

ARF: acute respiratory failure, CT-scan: computerized tomography-scan, HFNO: High flow nasal oxygen, LV: left ventricle, MV: Mechanical ventilation, NIV: Non-invasive ventilation, Pa02/Fi02: partial pressure of oxygen / fraction of inspired oxygen, RV: right ventricle, RBC: red blood cells, TAPSE: **t**ricuspid annular plane systolic excursion.

### Prognostic factors associated with ICU mortality

Univariate analysis identified high SAPSII (p<0.001), SOFA score (p = 0.001), low platelet count (p = 0.03), fungal infections (p = 0.04), MV requirement (p<0.001) and vasopressors use (p<0.001) associated with increased ICU mortality. The diagnosis of ARF with LVD (p = 0.01), interstitial pattern on chest CT-scan (p = 0.002), the use of NIV (p = 0.007), the use of diuretics (p = 0.001) and a negative hydric balance at 48 hours (p = 0<0.001) were associated with an increased ICU survival.

By multivariate analysis (Table 3), 3 factors were independently associated with an increased ICU mortality: SAPS II score (OR = 1.07/point, 95% CI = 1.03–1.11, p<0.001), invasive fungal infection (OR = 7.65, 95% CI = 1.7–34.6, p = 0.008) and initial ventilation strategy (p = 0.015). Compared to NIV (reference), HFNO alone and standard oxygen alone were associated with an increased ICU mortality, with respective OR of 19.56 (95% CI = 2–189.7, p = 0.01) and 10.72 (95% CI = 1.7–66.8, p = 0.01). Interestingly, significant positive-pressure ventilation modes (NIV associated with HNFO and MV) were not associated with an increased mortality, compared to insignificant-pressure ventilation (HNFO and oxygen).

**Table 3:** Multivariate analysis of factors associated with ICU mortality.

	Odds Ratio	95% CI	*p*
SAPSII (/point)	1.07	1.03–1.11	**<0.001**
Invasive fungal infection	7.65	1.7–34.6	**0.008**
Initial ventilation strategy			
NIV (reference)	1		**0.015**
HFNO	19.56	2.0–189.7	
Standard oxygen	10.72	1.7–66.8	
NIV + HFNO	3.02	0.5–18.1	
Immediate MV	2.44	0.35–17.1	

CI: confidence interval, HFNO: high flow nasal oxygen, ICU: intensive care unit, NIV: non-invasive ventilation, MV: mechanical ventilation, SAPSII: simplified acute physiology score.

### Propensity score

**Fig 2:**
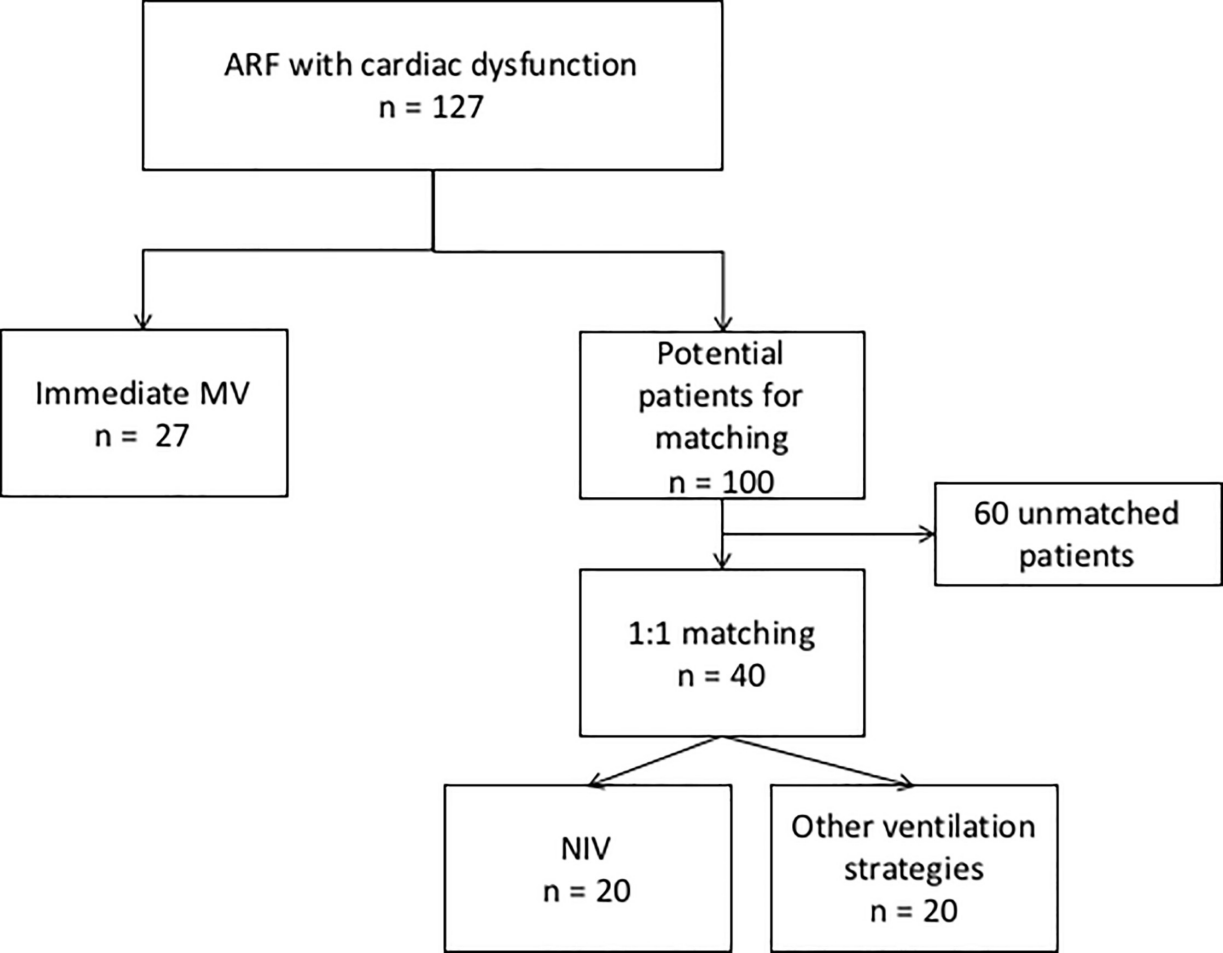
Flow chart of propensity score. MV: mechanical ventilation, NIV: non-invasive ventilation.

Propensity score compared 40 matched patients, 20 in the NIV arm and 20 receiving others ventilation strategies, excluding MV (Fig 2). All patients and ARF characteristics were comparable in both arms after matching (Table 4). ICU mortality rate was significantly lower in patients treated with NIV (10%, 2 patients), versus 50% (n = 10) in patients receiving other ventilation strategies (p = 0.037).

**Table 4:** Propensity score according to NIV use.

	NIV use (n = 20)	Absence of NIV (n = 20)	*p*	*Std diff*
Age	64 (59–71)	66 (57–70)	0.86	0.057
Gender (Female/Male)	9/11	7/13	0.48	0.204
Charlson comorbidity index	4 (2–7)	4 (2–8)	0.89	0.015
SAPSII at admission	45 (43–53)	47 (38–53)	0.96	0.035
SOFA score at admission	7 (5–11)	8 (6–10)	0.85	0.059
Neutropenia	5 (25)	8 (40)	0.37	0.309
Allogenic HSCT recipients	5 (25)	6 (30)	0.74	0.110
Sepsis at ICU admission	17 (85)	17 (85)	0.79	0.000
ARF diagnosis				
ARF with LVD	20 (100)	16 (80)	0.31	0.253
Infectious pneumonia	8 (40)	8 (40)	1	0.000
Atelectasia or pleural effusion	2 (10)	3 (15)	0.57	
Pulmonary embolism	1 (5)	2 (10)	0.57	
Vasodilators	8 (40)	5 (25)	0.27	0.301
Diuretics	16 (80)	16 (80)	1	0.000
Vasopressors at ICU admission	10 (50)	10 (50)	1	0.000
Noradrenaline	5 (25)	8 (40)	0.37	
Adrenaline	3 (15)	4 (20)	0.66	
Dobutamine	6 (30)	4 (20)	0.53	

ARF: acute respiratory failure, HSCT: hematopoietic stem cell transplantation, ICU: intensive care unit. LVD: left ventricular dysfunction, NIV: non-invasive ventilation, SAPSII: Simplified acute physiology score, SOFA: Sepsis-related Organ Failure Assessment, Std diff: Standardized differences.

## Discussion

ARF is the leading cause of ICU admission in cancer patients [1]. In this study, we assessed prognostic impact of initial ventilation strategy in onco-hematology patients presenting ARF with associated cardiac dysfunction. We identified severity score at ICU admission, invasive fungal infections and initial ventilation strategy as independently associated with ICU mortality.

We identified NIV as a protective factor on ICU mortality, compared with standard oxygen alone and HFNO alone. The absence of significant positive-pressure ventilation strategies (HNFO and standard oxygen) was associated with an increased mortality. The ventilation strategy in ARF has considerably evolved over the last decade and remains debated in immunocompromised patients. As mortality rate in patients requiring MV was up to 90% in the early 2000s, avoiding intubation became a priority and NIV was increasingly used in ARF besides its classical indications, with decreased intubation and mortality rates [3]. However, mortality rate in the control group was extremely high (90%) in Hilbert publication [3]. Outcome of critically ill cancer patients has greatly improved over the last years [20, 21], including in patients with severe ARDS and in non-ARDS patients receiving MV. The recent literature is equivocal in terms of harm or benefit from NIV. Hilbert’s results have not been confirmed by recent studies and NIV effectiveness for hypoxemic ARF in cancer patients remains unclear. Overall while this study holds interesting results, it is an observational retrospective study in a specialized center that may have difficulty with generalizability in regards to change in care. In the recent FLORALI study, Frat compared NIV versus HFNO versus standard oxygen in hypoxemic ARF in a multicenter prospective trial. There was no difference of intubation rate in the 3 groups, but ICU mortality rate, which was a secondary outcome, was significantly higher in the NIV group [12]. Frat confirmed the deleterious role of NIV in immunocompromised patients, with increased intubation and mortality rates in patients treated with NIV compared to those treated with HFNO [13]. However, NIV settings were controversial. In the randomized controlled iVNIctus trial, assessing early NIV in immunocompromised patients with hypoxemic ARF, NIV was compared to standard oxygen, with no difference in mortality rate, oxygenation failure, ICU-acquired infections, duration of MV or lengths of ICU or hospital stays [19]. In the recently published EFRAIM trial, HFNO has an effect on intubation but not on mortality rates. Moreover, failure to identify ARF etiology was associated with higher rates of both intubation and mortality, enhancing the crucial role of identifying ARF diagnostic [27]. The recent development of HFNO may have help to improve outcomes and raised concerns about NIV [12, 13, 28]. We reported significant improvement of day-28 mortality in cancer patients with ARF treated with HFNO associated with NIV (HFNO-NIV), as compared with other patients (HFNO alone, NIV with standard oxygen, standard oxygen alone). After propensity score analysis, HFNO-NIV was associated with improved survival, decreased ventilator-free days and decreased day-28 mortality [15].

Taken together, these results strongly suggest that early NIV does not translate anymore into survival benefits and could be harmful. In ARF with severe hypoxemia, NIV should be avoided, because half the patients with severe ARF and 75% of severe ARDS patients experienced NIV failure with significantly higher mortality [29]. To date, no study has evaluated NIV versus intubation in patients meeting the indications for intubation as performed in unselected patients.

While NIV has been validated in acute cardiac pulmonary edema [23], the optimal ventilation strategy in mixed ARF is still unknown, particularly in immunocompromised patients. In our study, NIV seemed interesting in this situation, alone or associated to HNFO. The absence of significant positive-pressure ventilation (HFNO alone and standard oxygen alone) is associated with an increased mortality. Interestingly, using propensity score analysis the mortality rate of patients treated with NIV was about 10%, which is comparable with mortality rates reported in the general population treated for ACPE [23]. Mixed ARF are probably under evaluated in cancer patients, who frequently develop ARF in context of hydric inflation, transfusions, sepsis, and worsened by cardiac toxicity of anthracyclines or targeted therapies and chronic anemia.

We also confirmed organ dysfunction severity at ICU admission and invasive fungal infections as adverse prognostic factors [1, 6]. Our results are in line with literature. In a recent study, the diagnosis of invasive fungal infection was the most relevant early predictive factors of the severity of ARDS in hematology patients [30]. In another cohort of cancer patients with ARDS, invasive fungal infection was identified as a risk factor for higher mortality [6].

This study has several limitations. First, its retrospective nature is intrinsically susceptible to have selection bias; however all biological and medical parameters were collected prospectively with our information system. Second, it is a monocentric study in a specialized center accustomed to the ICU management of cancer patients presenting with ARF, these results must therefore be analyzed with caution and cannot be generalized to other centers. Third, there is no data in the literature concerning prognosis of cancer patients admitted to the ICU for ARF with associated cardiac dysfunction, so the external validity of our study must be confirmed. Finally, extracting medical information coding was not our main strategy for selecting eligible patients for this study. It was therefore impossible for us, given the patient selection methodology, to assess the specificity of the patient selection methodology.

## Conclusions

This study demonstrated a benefit of NIV in a cohort of critically cancer patients admitted to the ICU for ARF with associated cardiac dysfunction. In these patients, NIV still has a place in the ventilation strategy, despite recent conflicting data and probably represents a good indication. These clinical situations are probably under evaluated. Unanswered questions remain on optimal ventilation strategy in critically ill cancer patients admitted to the ICU for hypoxemic ARF. Further studies are needed to address this issue according to ARF severity and etiology, in order to identify the best indications for each ventilatory support.
